# PAX5-induced upregulation of IDH1-AS1 promotes tumor growth in prostate cancer by regulating ATG5-mediated autophagy

**DOI:** 10.1038/s41419-019-1932-3

**Published:** 2019-09-30

**Authors:** Nan Zhang, Zhongyi Li, Fuding Bai, Shigeng Zhang

**Affiliations:** Department of Urology, The Second Affiliated Hospital Zhejiang University School of Medicine, No.88 Jiefang Road, 310009 Hangzhou, China

**Keywords:** Prostate cancer, Cell biology

## Abstract

Prostate cancer (PCa) is one of the major malignancies affecting males’ health around the world. Long noncoding RNAs (lncRNAs), a class of long transcripts, has been reported as essential regulators in tumorigenesis. IDH1 antisense RNA 1 (IDH1-AS1) is an lncRNA which can interact with genes to regulate the Warburg effect. However, function and mechanism of it in tumorigenesis of PCa remains unclear. Therefore, our current study focused on exploring the role of IDH1-AS1 in PCa tumor growth. At first, the expression of IDH1-AS1 was identified to be upregulated in PCa samples and cell lines. Mechanism associated with the upregulation of IDH1-AS1 was analyzed and demonstrated by mechanism experiments. The result suggested that PAX5 is the transcriptional activator of IDH1-AS1. Functionally, loss-of function assays revealed that silencing of IDH1-AS1 inhibited cell proliferation and induced cell apoptosis both in vitro and in vivo. Through microarray analysis and Gene ontology (GO) analysis, we determined that IDH1-AS1 can affect PCa cell autophagy by upregulating ATG5 expression. Mechanism investigation further validated that IDH1-AS1 posttranscriptionally regulated ATG5 expression by enhancing the mRNA stability of ATG5 or upregulating ATG5 by sequestering miR-216b-5p. Consequently, rescue assays demonstrated that IDH1-AS1 promoted proliferation and apoptosis in PCa via ATG5-induced autophagy. Taken together, our study elucidated the function and regulatory mechanism of IDH1-AS1, thus providing a novel biomarker for PCa.

## Introduction

Prostate cancer (PCa), one of the most common diagnosed male malignancies^1^. Lacking of diagnostic biomarker in early stage is the main cause of low overall survival rate of patients with PCa. Despite the efforts in exploration of novel therapeutic strategies, patients’ prognosis remains unfavorable. Thus, investigation of molecular mechanism involved in the tumorigenesis of PCa is essential to finding novel diagnostic biomarkers.

Long noncoding RNAs (lncRNAs), are a type of transcripts with length over 200 nt, which have become the research focus in cancer-associated transcriptome^2^. Functionally, lncRNAs can serve as biological participants in diverse processes^3,4^. In addition, lncRNAs have been characterized as oncogenes or tumor suppressors in tumorigenesis^5–7^. Recently, PCa-specific lncRNAs were also defined^8,9^. In this study, we screened lncRNAs associated with PCa from online database. IDH1 antisense RNA 1 (IDH1-AS1) is a lncRNA that was differentially expressed in PCa samples obtained from database or 62 PCa patients. Xiang S et al. has reported the potential function of IDH1-AS1 in cancer growth^10^. Nevertheless, it is unclear whether IDH1-AS1 exerted function in PCa tumor growth via regulating molecular mechanism.

Transcription activation can induce upregulation of lncRNAs, thus promoting tumorigenesis^11–13^. Here, we also detected the transcription regulator of IDH1-AS1 through bioinformatics analysis and mechanism investigation. Moreover, loss-of function study was carried out in PCa cells to determine the role of IDH1-AS1 in regulating PCa cell growth both in vitro and in vivo. Downstream molecular mechanism of IDH1-AS1 was analyzed using microarray analysis and GO analysis. Then, we further detected whether IDH1-AS regulated PCa cell growth via ATG5-induced autophagy. The regulatory pattern of IDH1-AS1 in ATG5 was assessed by mechanism experiments.

LncRNAs are acknowledged to act as posttranscriptional regulators in tumorigenesis by serving as competing endogenous RNAs^14–16^ or interact with RNA-binding proteins to enhance mRNA stability^17–20^. In this regard, we investigated the posttranscriptionally regulatory effect of IDH1-AS1 on ATG5. Collectively, the focus of this study is on exploring the role of PAX5-activated IDH1-AS1 in PCa tumor growth via ATG5-induced autophagy.

## Materials and methods

### Bioinformatics analysis

The expression pattern of IDH1-AS1 in human normal tissues and PCa tissues was downloaded from TCGA dataset (http://gepia.cancer-pku.cn/index.html). The DNA motif of PAX5 and its putative binding sites within IDH1-AS1 promoter were predicted via JASPAR tool (http://jaspar.genereg.net/).

### Patients and clinical specimens

A total of 62 pairs of PCa and corresponding para-cancerous tissues were randomly acquired from patients who underwent surgical resection at the Second Affiliated Hospital Zhejiang University School of Medicine from January 2012 to December 2018. All experimental subjects had not received any other treatment prior to operation. Written informed consents were signed by all subjects for study purpose. Our study was approved and supervised by The Ethics Committee of the Second Affiliated Hospital Zhejiang University School of Medicine.

### Cell lines and culture

Human prostate epithelial cell line (RWPE-1) and four acknowledged human PCa cell lines (PC-3, DU145, VCaP, and LNCaP) were all acquired commercially from the American Type Culture Collection (ATCC, Manassas, VA, USA). All cell lines were kept in RPMI 1640 medium (Gibco-BRL, Carlsbad, CA) with 10% fetal bovine serum. Cell culture was performed in a humidified incubator with 5% CO_2_ at 37 °C.

### RNA isolation and quantitative real-time PCR (qRT-PCR)

At first, total RNAs were isolated from cells using Trizol reagent (Invitrogen, Carlsbad, CA, USA) following the user guide. Reverse transcription was conducted using the Applied Biosystems™ High-Capacity cDNA Reverse Transcription Kit. SYBR^®^ Green Real-Time PCR Master Mixes (Thermo Fisher Scientific, Waltham, MA, USA) was used to prepare PCR amplification reaction with GAPDH as endogenous control. For detecting miR-216b-5p level, miRNA was isolated using the mirVana miRNA Isolation Kit (Thermo Fisher Scientific), reverse transcription was conducted by the miScript II RT Kit (QIAGEN, Inc., Valencia, CA, USA), and PCR reaction was prepared by the mirVana qRT-PCR miRNA Detection Kit (Thermo Fisher Scientific) with U6 as endogenous control. All targets and references were amplified in triplicate. The relative expression levels of targets were calculated using 2^−△△Ct^ method.

### Cell transfection and plasmids

VCaP and LNCaP cell lines were cultivated all night to reach 70–80% confluence. All cell transfections were operated by use of lipofectamine2000 (Thermo Fisher Scientific). The pcDNA3.1 vector expressing PAX5, PTBP3, or ATG5 and empty vector (named as Ctrl) were designed and constructed by Sangon (Shanghai, China). To knockdown the expression of PAX5 or IDH1-AS1, the short hairpin RNAs (shRNAs) against PAX5 or IDH1-AS1 (named as shPAX5 or shIDH1-AS1#1/2) and non-specific shRNA (named as shCtrl) were synthesized by GenePharma (Shanghai, China). MISSION^®^ microRNA Mimics miR-216b-5p and scrambled negative control (named as miR-NC) were from Sigma-Aldrich (St. Louis, MO, USA). After 48 h of incubation, cells were reaped from three independent experiments and used for subsequent analysis.

### Luciferase reporter assay

IDH1-AS1 promoter was amplified by PCR and subcloned into the pGL3-Basic vector (Promega, Madison, WI, USA), VCaP and LNCaP cell lines were seeded in 96-well culture plates (8000 cells/well) and co-transfected with reporter plasmids containing IDH1-AS1 promoter, pRL-TK-Renilla plasmid (Promega, USA) and pcDNA-PAX5 or shPAX5, along with their respective control (Ctrl or shCtrl). After 48 h, luciferase activity was measured using the Dual-Luciferase Reporter Assay System (Promega, USA). For assessing the interaction between miR-216b-5p and IDH1-AS1 or ATG5, cells were co-transfected with miR-216b-5p mimics or miR-NC, together with pmirGLO-IDH1-AS1-WT/Mut vector (Sangon, China) or psiCheck2-ATG5-WT/Mut vector (Generay Biotech, Shanghai, China). Biological replications were conducted for three times.

### Chromatin immunoprecipitation (ChIP) assay

ChIP assay was conducted by use of the EZ ChIP™ Chromatin Immunoprecipitation Kit for cell line (Millipore, Bedford, MA, USA). VCaP and LNCaP cell lines were first fixed in 1% formaldehyde for 15 min crosslink. Thereafter, crosslinked chromatin was ultra-sonicated into 200- to 1000-bp fragments, and then immunopreciated with anti-PAX5 or control anti-IgG bound-protein G beads for 6 h. qRT-PCR was used to evaluate the enrichments of DNA fragments immunoprecipitated by antibodies. ChIP assay was performed in triplicate.

### Cell proliferation assays

VCaP and LNCaP cell lines were seeded into 96-well plates (1.0 × 10^3^ cells/well) in triplicate. Ten microliters of the Cell Counting Kit-8 (CCk-8, Dojindo, Kumamoto, Japan) was added into each well and incubated for 2 h in line with the user guide. Cell ability was examined by measuring the absorbance at 450 nm every 24 h for 4 days.

For colony formation assay, cells were seeded at a density of 500 cells per well into six-well plates, followed by incubation for two weeks. The colonies were dyed with 0.5% crystal violet solution for 15 min and finally counted.

For EdU incorporation assay, the Cell-light™ EdU ApolloR567 In Vitro Imaging Kit was purchased from Ribobio (Guangzhou, China). Cells were transfected and planted into 96-well culture plates (8000/well) all night. After 2 days, cells were cultivated with 25 μM of Edu for 4 h and fixed in 4% paraformaldehyde for 30 min, following permeabilizing with 0.5% TritonX-100 for 10 min. DAPI was utilized to stain the total cells. All proliferative assays were repeated independently for at least three times.

### Cell apoptosis assays

Cell apoptosis was measured by JC-1 assay. VCaP and LNCaP cell lines were put into 96-well culture plates at the density of 1.0 × 10^4^ cells per well and cultivated overnight. Following centrifugation at 1000 rpm for 5 min at room temperature, culture medium was removed and cells were loaded with JC-1 dye for half an hour. After washing, the change in mitochondrial transmembrane potential (ΔΨ_m_) was detected with a fluorescent plate reader. Images were captured by a fluorescence microscope.

For TUNEL staining assay, cells were transfected and cultivated all night. After rinsing twice in phosphate-buffered saline (PBS), cells were fixed in 4% paraformaldehyde for 15 min and permeabilized in 0.25% Triton‐X 100 for 20 min. TUNEL staining method was used following the guidelines of In Situ Cell Death Detection Kit (Roche Molecular Diagnostics, Pleasanton, CA, USA). Cell nuclei were treated with DAPI. Positively stained cells were observed under an EVOS FL microscope (Thermo Fisher Scientific, Waltham, MA, USA).

For detection of caspase-3 activity, caspase-3 activity kit was bought from Solarbio (Beijing, China). Cell proteins were extracted and seeded into 96-well plates. Afterwards, proteins were incubated with caspase-3 substrate for 3 h. The absorbance was detected at the wavelength of 405 nm. All results of apoptotic assay were obtained from three different replications.

### In vivo tumorigenesis assay

Male nude mice, aged ~6 weeks, were kept in an SPF-grade pathogen-free animal laboratory. This animal study had obtained the approval of the Animal Research Ethics Committee of the Second Affiliated Hospital Zhejiang University School of Medicine. A total of 5 × 10^6^ LNCaP cells were transfected and subcutaneously injected around the left flank of the nude mice (five mice per group). Tumor volume was examined as 0.5 × length × width^2^ every 7 days. Four weeks later, mice were killed, tumors were excised and weighed for further study.

### Microarray analysis

Cells were transfected with shRNAs against IDH1-AS1 or negative control. Total RNA was extracted after 48 h transfection. Gene expression microarray analysis (Agilent Technologies, Santa Clara, CA, USA) was carried out as per the user guide. One hundred nanograms of total RNA was amplified and labeled by the Low-input Quick Amp Labeling Kit One-color (Agilent Technologies). The synthesized cRNA was hybridized to the SurePrint G3 Human GE microarray v2 (G4851; Agilent Technologies). Finally, the differentially expressed mRNAs were identified with the cut-off criteria of *P* < 0.05 and fold-change >2. In our present study, Gene ontology (GO) analysis was performed based on the GO database (http://www.geneontology.org/). *P* < 0.05 was considered to indicate statistical significance.

### Western blot assay

Cell protein samples were extracted using RIPA buffer reagent (Thermo Fisher Scientific) and separated using 12% SDS PAGE gels. Following transferring onto polyvinylidene fluoride membranes, bovine serum albumin (5%) solution (Beyotime, Guangzhou, China) was used to block membranes for 2 h at room temperature. Primary antibodies, including anti-LC3 I/II (ab128025, Abcam, Cambridge, USA), anti-p62 (ab56416, Abcam), anti-Beclin1 (ab210498, Abcam), anti-ATG5 (ab228668, Abcam), and anti-GAPDH (ab181602, Abcam), were incubated with membranes at 4 °C all night. Secondary antibodies were cultured with membranes at room temperature for 2 h. At length, bound proteins were quantified using ECL Prime Western Blotting Detection reagent (GE Healthcare, Chicago, IL, USA). All experimental procedures were repeated for more than two times.

### Immunofluorescence assay

VCaP and LNCaP cell lines were plated on culture slides until adherent to the slides. After rising in PBS twice, cells were fixed in ice-cold methanol-acetone for 10 min and sealed in 5% BSA for 10 min. Subsequently, cells were cultured with the primary antibodies against LC3 for 2 h at room temperature and rinsed in PBS for three times. Then, cells were incubated with secondary antibodies for 1 h. After staining with DAPI for 10 min, the slides from three replications were observed using a fluorescence microscope (Olympus, Tokyo, Japan).

### MDC staining assay

VCaP and LNCaP cell lines were transfected and seeded in six-well plates for 3 days and rinsed for three times in PBS. Monodansylcadaverine solution (MDC; Sigma-Aldrich) was diluted to 1:10 and added staining solution to cover cells. Three days later, culture medium was discarded, the plates were washed twice with 300 µl wash buffer. Then, cells were mixed with 50 µmol/l at 37 °C for 1 h in the dark. Following fixation in 4% paraformaldehyde for 15 min, cells were washed twice in cold PBS and photographed by fluorescence photometry. Three different experimental results were determined at 512 nm.

### RNA immunoprecipitation (RIP) assay

For RNA-binding protein immunoprecipitation assay, EZ-Magna RIP kit (Millipore) was utilized in line with the guidelines supplied by the manufacturer. Cell lysates were cultured with RIP buffer containing magnetic beads conjugated to human anti-PTBP3 antibody (Cell Signaling Technology, Danvers, MA) or negative control normal mouse IgG antibody (Millipore, CA, USA). Anti-SNRNP70 antibody was seen as the positive control. Lysates were treated with Proteinase K and finally subjected to silver staining and qRT-PCR analysis. For Ago2-RIP assay, cells were incubated with protein A/G sepharose beads conjugated to antibodies against Ago2 (Millipore, Massachusetts, USA) or IgG. Immunoprecipitated RNAs were extracted for qRT-PCR. Experiments were performed in triplicate.

### Subcellular fractionation assay

Cytoplasmic and nuclear RNA were isolated based on the user guide of PARIS™ Kit (Invitrogen, USA). A total of 1.0 × 10^7^ VCaP and LNCaP cell lines were lysed in cell fractionation buffer and incubated on ice for 10 min. After centrifugation, the upper solution was discarded, the nuclear pellet was collected to extract RNA in cell disruption buffer. Biological triplicates were carried out and followed by qRT-PCR to detect RNA abundance.

### Statistical analysis

All statistical analysis was conducted by SPSS version 20.0 software (IBM Corp., Armonk, NY, USA). Values were presented as the mean ± standard deviation. *P*-value threshold was set as 0.05 to indicate a statistically significant difference. Statistical comparisons were carried out using the Student’s *t* test or one-way analysis of variance followed by the Dunnett’s test. Gene correlations were analyzed by the Pearson’s correlation method.

## Results

### IDH1-AS1 is upregulated in PCa tissues and cell lines

**Fig. 1:**
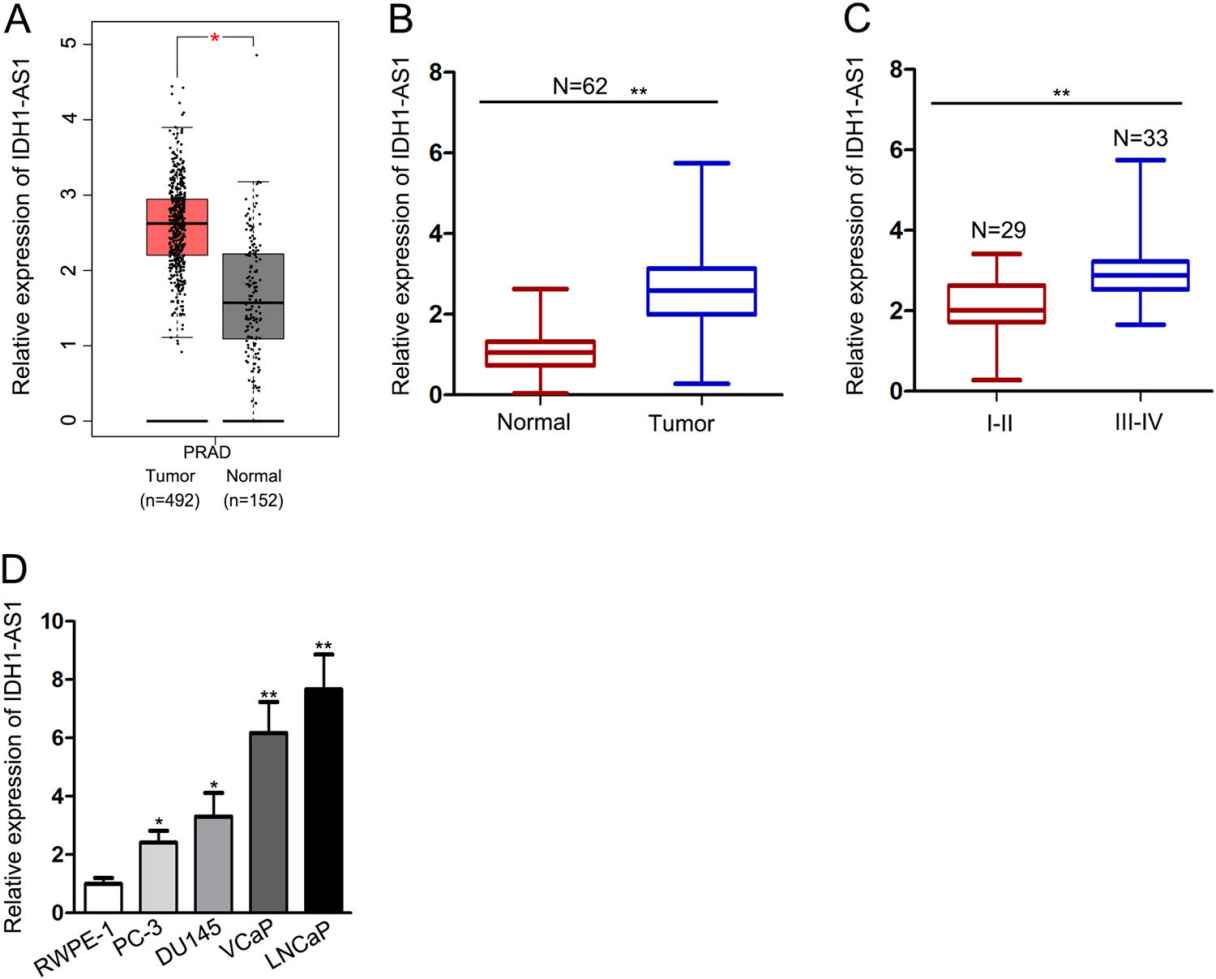
IDH1-AS1 is upregulated in PCa tissues and cell lines. **a** IDH1-AS1 expression level in PCa samples obtained from GEPIA database. **b** qRT-PCR showing IDH1-AS1 expression in paired PCa and normal samples collected from 62 PCa patients. **c** Expression level of IDH1-AS1 in PCa tissues in early stage of advanced stage was measured using qRT-PCR analysis. **d** IDH1-AS1 expression level was examined in PCa cell lines and normal epithelial cell line. *P < 0.05, **P < 0.01

According to the gene expression profile in GEPIA database, IDH1-AS1 is a highly expressed lncRNA in PCa samples (Fig. 1a). Sixty-two PCa patients were recruited in this study. And these patients were divided into two groups in accordance with tumor stage (I/II and III/IV). IDH1-AS1 expression was detected in different tissues collected from these patients. After qRT-PCR exanimation, we determined that IDH1-AS1 was expressed higher in tumor tissues and advanced stage patient samples compared with normal tissues and early-stage patient samples (Fig. 1b, c). Consistently, IDH1-AS1 expressed at a higher level in PCa cell lines compared with normal epithelial cell line (Fig. 1d). These data suggested that IDH1-AS1 potentially participated in tumorigenesis of PCa.

### IDH1-AS1 is transcriptionally activated by PAX5 in PCa

**Fig. 2:**
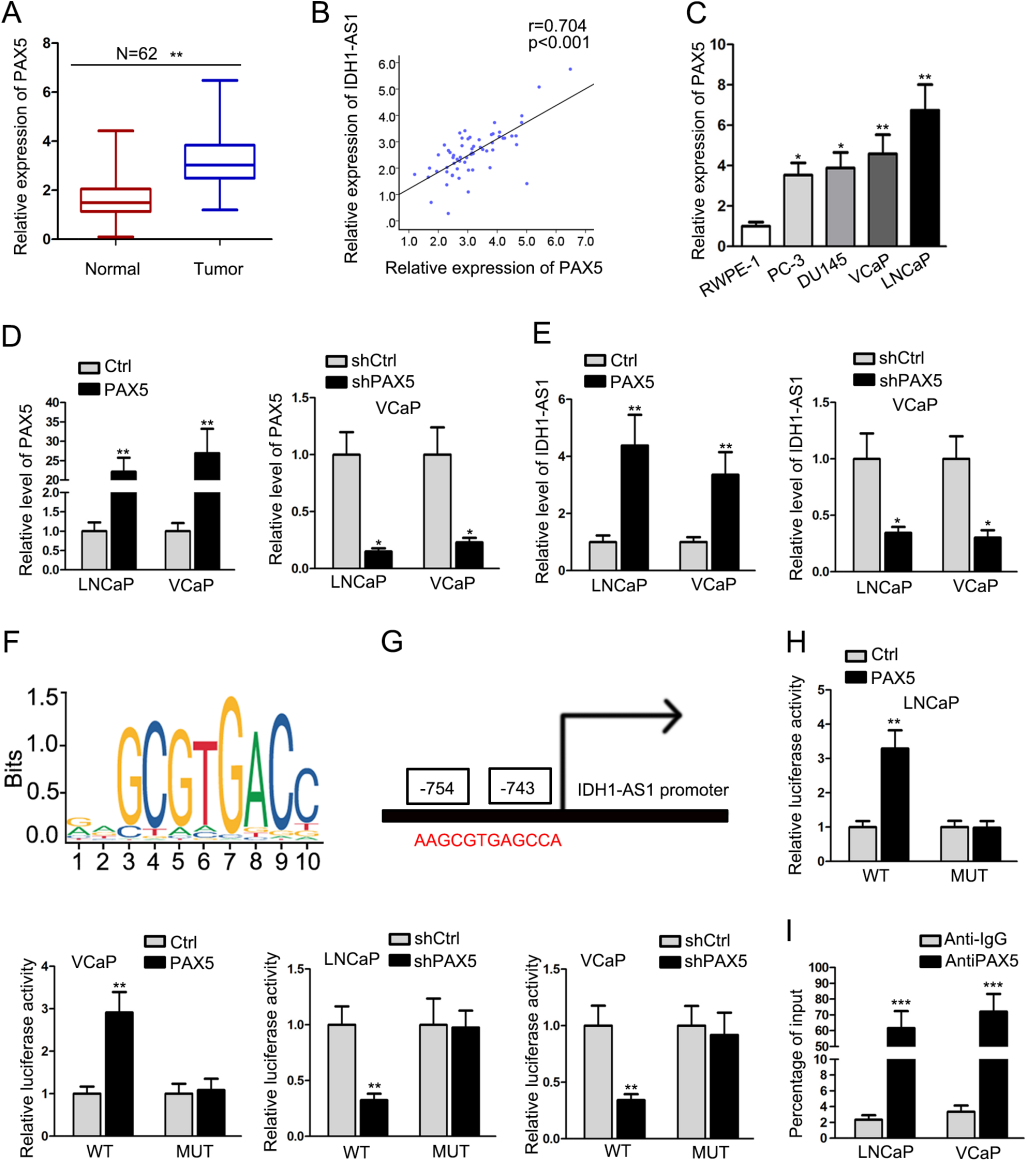
IDH1-AS1 is transcriptionally activated by PAX5 in PCa. **a** PAX5 expression was assessed in 62 PCa samples and paired normal tissues. **b** Expression association between PAX5 and IDH1-AS1 in PCa tissues was analyzed by the Pearson correlation test. **c** Relative PAX5 expression in PCa cell lines and normal prostate epithelial cell line. **d** PAX5 was silenced or overexpressed it in LNCaP and VCaP cells. Transfection efficiency was determined by qRT-PCR. **e** The expression changes of IDH1-AS1 in PAX5-upregulated or -downregulated cells were detected. **f** DNA-binding motif of PAX5 was obtained from IASPAR. **g** Predicted binding sites of PAX5 in the promoter region IDH1-AS1. **h** pGL3 luciferase reporter assay was conducted to demonstrate the interaction between PAX5 and IDH1-AS1 promoter. **i** The affinity of PAX5 in the promoter region of IDH1-AS1 was assessed by ChIP assay. *P < 0.05, **P < 0.01

Transcription activation is one of the main molecular mechanisms which led to the upregulation of genes. Screening from UCSC online database, PAX5 is a potential upstream transcription regulator for IDH1-AS1. According to qRT-PCR analysis, PAX5 was identified to upregulated in 62 PCa samples (Fig. 2a), which had a positive expression correlation with IDH1-AS1 (Fig. 2b). Similarly, PAX5 was also expressed at a relative high level in PCa cell lines (Fig. 2c). To assess whether PAX5 had regulatory effect on IDH1-AS1 expression, we silenced or overexpressed it in LNCaP and VCaP cells (Fig. 2d). Then, we examined the expression change of IDH1-AS1 in above cell lines and uncovered the positive regulatory effect of PAX5 on IDH1-AS1 expression in PCa cells (Fig. 2e). DNA binding motif of PAX5 was obtained from JASPAR (Fig. 2f) and was subjected to the prediction of binding sites with IDH1-AS1 promoter. There existed a binding sequence between PAX5 and IDH1-AS1 promoter (Fig. 2g). Next, pGL3 luciferase reporter assay was conducted to demonstrate whether the binding sequence was responsible for the interaction between PAX5 and IDH1-AS1 promoter. Data presented in Fig. 2h, the luciferase activity of IDH1-AS1 promoter was increased after overexpression of PAX5 but was reduced in cells transfected with shPAX5. Furthermore, we found that PAX5 had a strong affinity in the promoter region of IDH1-AS1 through ChIP assay (Fig. 2i). Collectively, upregulation of IDH1-AS1 in LUAD cell lines might be caused by PAX5-induced transcription activation.

### IDH1-AS1 acts as a facilitator for PCa cell growth in vitro and in vivo

**Fig. 3:**
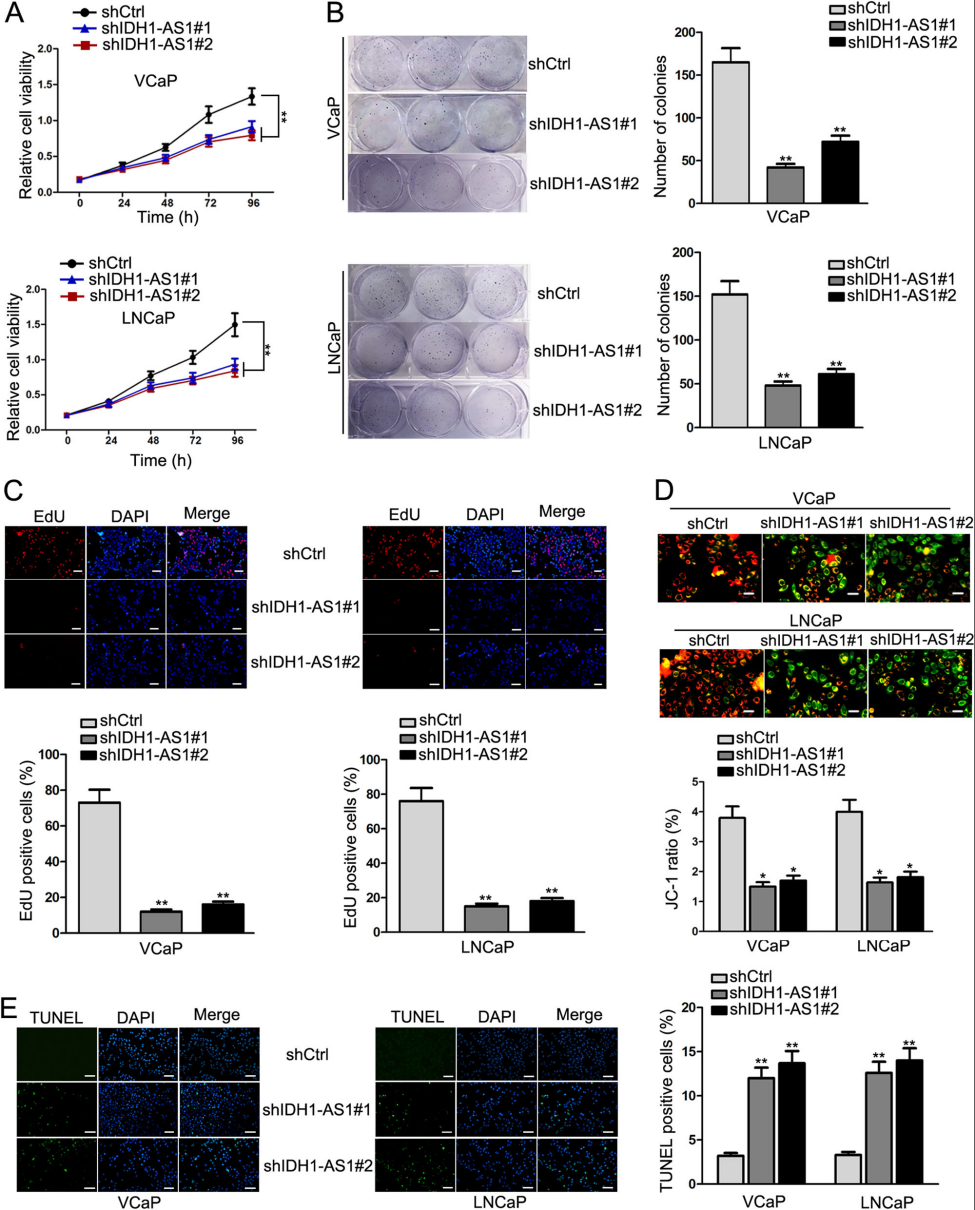
IDH1-AS1 acts as a facilitator for PCa cell growth. **a**–**c** Proliferative ability of LNCaP and VCaP cells transfected with shCtrl, shIDH1-AS1#1 or shIDH1-AS1#2 was evaluated by CCK-8 (**a**), colony formation (**b**) and EdU assays (**c**). **d**, **e** Apoptosis was measured in transfected PCa cells via JC-1 (**d**) and TUNEL assays (**e**). *P < 0.05, **P < 0.01. Scale bars for EdU, TUNEL, JC-1 assays equal 200 μm

Based on findings above, we determined the upregulation of IDH1-AS1 in PCa and its upregulatory mechanism. Considering the highest expression level of IDH1-AS1 in VCaP and LNCaP cells, we conducted loss-of-function studies in these two cells. Silencing of IDH1-AS1 by shRNAs was determined by qRT-PCR analysis (Supplementary Fig. 1a). At first, we evaluated the effect of silenced IDH1-AS1 on cell proliferation. Through cell proliferation assays, including CCK-8, colony formation and EdU assays, we uncovered that cell proliferation was attenuated in response to the downregulation of IDH1-AS1 (Fig. 3a–c). In addition, apoptosis was measured in transfected PCa cells via JC-1 and TUNEL assays. As expected, suppression of IDH1-AS1 expression accelerated apoptosis (Fig. 3d, e). For further evidence, animal study was carried out. After 28 days, tumors were removed from the body of nude mice. After observation and calculation, we identified that tumors in the shIDH1-AS1 group grew smaller than those in the shCtrl group (Supplementary Fig. 1b). This tendency was further validated in tumor volume and weight (Supplementary Fig. 1c, d). Based on all these data, IDH1-AS1 was identified as a tumor facilitator in PCa.

### IDH1-AS1 regulated ATG5-mediated autophagy in PCa

**Fig. 4:**
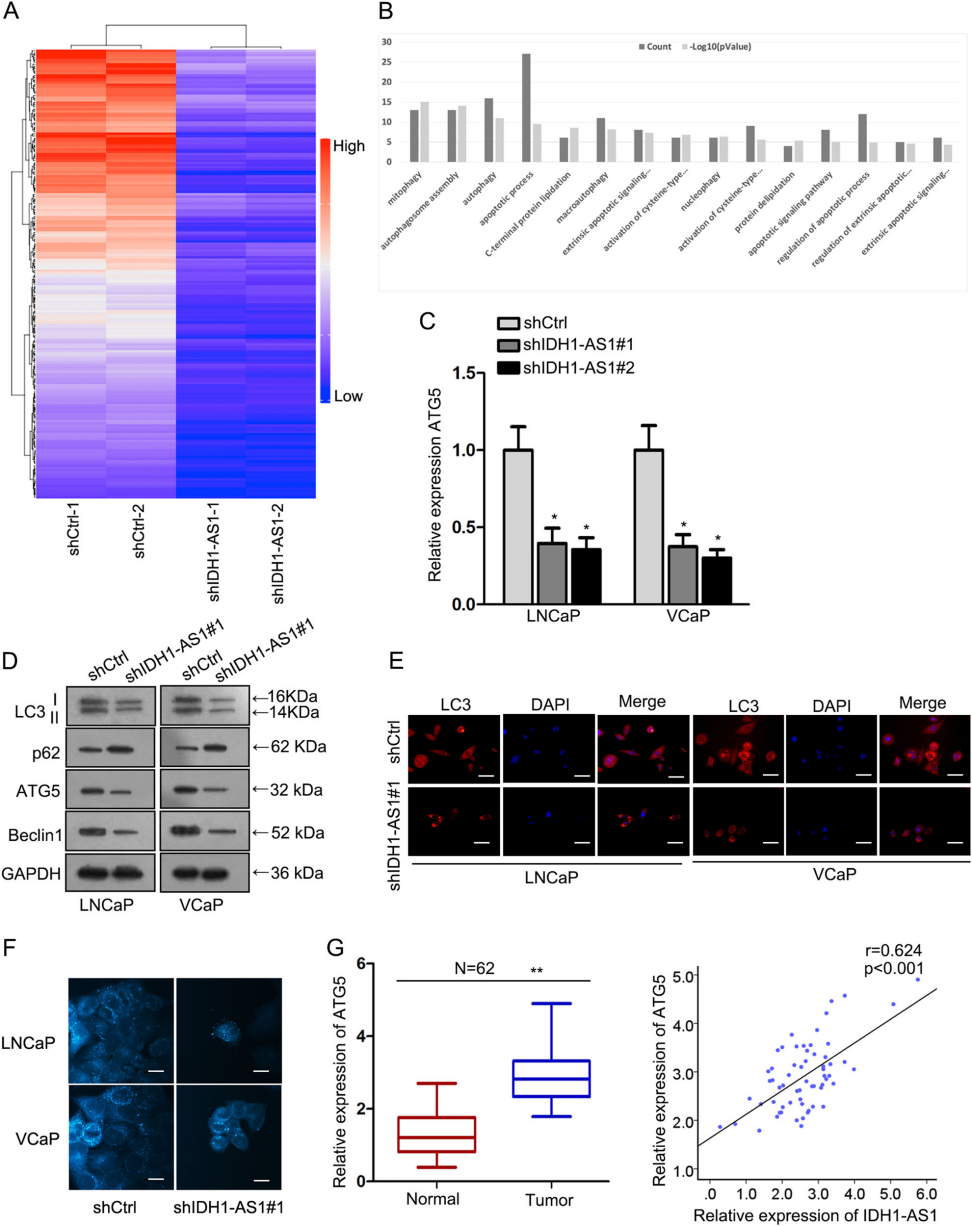
IDH1-AS1 regulated ATG5-mediated autophagy in PCa. **a** Top 200 mRNAs that were significantly downregulated in IDH1-AS1-silenced cells were screened out by microarray analysis. **b** GO analysis of IDH1-AS1-reulated 200 mRNAs. **c** ATG5 expression level in cells transfected with IDH1-AS1-specific shRNAs and control shRNA was measured using qRT-PCR examination. The level of autophagy-related proteins, LC3 fluorescence intensity and the ratio of autophagosome were detected successively by western blotting (**d**), LC3 immunofluorescence (**e**), and MDC staining (**f**). **g** ATG5 expression in PCa tissues and its expression association with IDH1-AS1 expression. *P < 0.05, **P < 0.01. Scale bars for LC3 immunofluorescence and MDC staining equal 200 μm

Subsequently, we analyzed IDH1-AS1-dominated molecular mechanism in PCa. Through microarray analysis, we screened out top 200 mRNAs that were significantly downregulated in IDH1-AS1-downregulated cells (Fig. 4a). Then, these 200 mRNAs were subjected to GO analysis. As presented in Fig. 4b, IDH1-AS1-dominated molecular mechanism potentially mediated cell apoptosis and autophagy. Importantly, ATG5, a biomarker for autophagy, belongs to top 200 downregulated mRNAs. Using qRT-PCR examination, we further demonstrated the positive regulation of IDH1-AS1 on ATG5 in two PCa cells (Fig. 4c). Thus, we further detected whether IDH1-AS1 regulated ATG5-mediated autophagy in PCa cells. The level of autophagy-related proteins, LC3 fluorescence intensity, and the ratio of autophagosome were detected successively. All experimental results indicated that IDH1-AS1 acted as an inducer in autophagy of PCa cells (Fig. 4d, f). Finally, ATG5 expression was uncovered to be higher in PCa tissues than in normal tissues, which had a consistent tendency with IDH1-AS1 expression (Fig. 4g). Collectively, IDH1-AS1 might regulate autophagy through ATG5.

### IDH1-AS1 interacted with PTBP3 to stabilize ATG5 mRNA

**Fig. 5:**
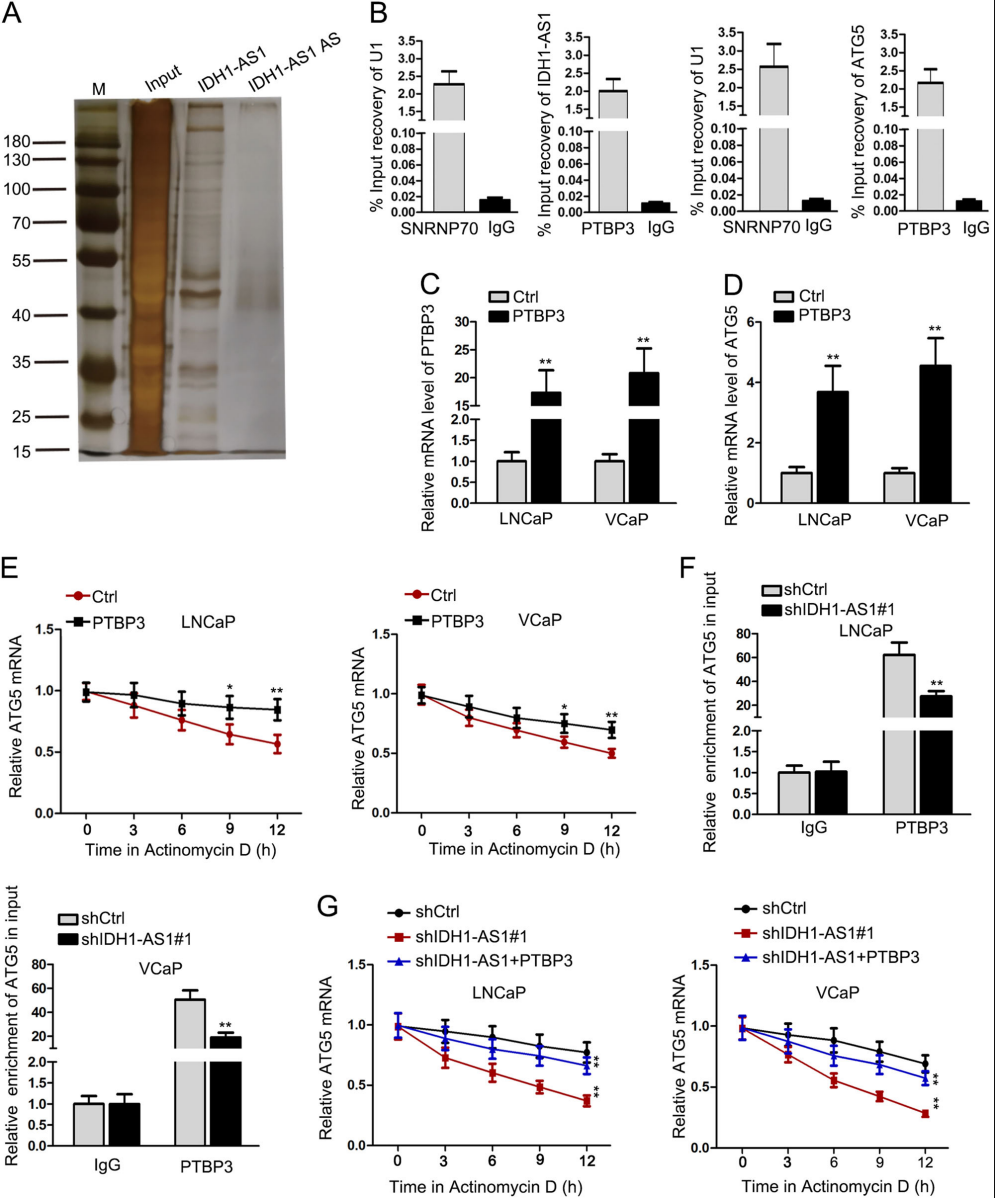
IDH1-AS1 interacted with PTBP3 to stabilize ATG5 mRNA. **a** Pull-down silver staining showing proteins which could interact with IDH1-AS1. **b** The interaction between PTBP3 and IDH1-AS1 or ATG5 was proved by RIP assay. **c**, **d** Overexpression of PTBP3 in LNCaP and VCaP cells and relative ATG5 expression were detected by qRT-PCR examination. **e** ATG5 mRNA stability was assessed in PCa cells treated with actinomycin D after overexpression of PTBP3. **f** RIP assay was applied to detect the interaction between ATG5 and PTBP3 in PCa cells after silencing of IDH1-AS1. **g** ATG5 mRNA stability was evaluated in cells transfected with shIDH1-AS1 or co-transfected with shIDH1-AS1 + PTBP3. *P < 0.05, **P < 0.01

To determine the regulatory pattern of IDH1-AS1 in PCa cells, we analyzed its cellular localization. The results shows in Supplementary Fig. 1e indicated that IDH1-AS1 was predominantly located in cytoplasm of PCa cells. Results of pGL3 luciferase reporter assay revealed that there was no significant effect of IDH1-AS1 on the luciferase activity of ATG5 promoter (Supplementary Fig. 1f), which eliminated the transcriptional regulation of IDH1-AS1 on ATG5. Through RNA pull-down and mass-spectrometry analysis, we found that PTBP3 is a RBP which could interact with IDH1-AS1 (Fig. 5a). The interaction between PTBP3 and IDH1-AS1 or ATG5 was further proved by RIP assay (Fig. 5b). Then, we overexpressed PTBP3 in LNCaP and VCaP cell and found that ATG5 expression was observably increased (Fig. 5c, d). Subsequently, ATG5 mRNA stability was assessed in PCa cells treated with Actinomycin D after overexpression of PTBP3. It was found that PTBP3 overexpression enhanced the stability of ATG5 (Fig. 5e). Furthermore, we analyzed whether IDH1-AS1 affected the interaction between PTBP3 and ATG5. As a result, silencing of IDH1-AS1 attenuated the interaction between PTBP3 and ATG5 (Fig. 5f). Accordingly, knockdown of IDH1-AS1 impeded the mRNA stability of ATG5, which tendency was attenuated after co-transfection with PTBP3 expression vector (Fig. 5g). Taken together, IDH1-AS1 regulated ATG5 mRNA stability via interacting with PTBP3.

### IDH1-AS1 upregulated ATG5 expression by sponging miR-216b-5p

**Fig. 6:**
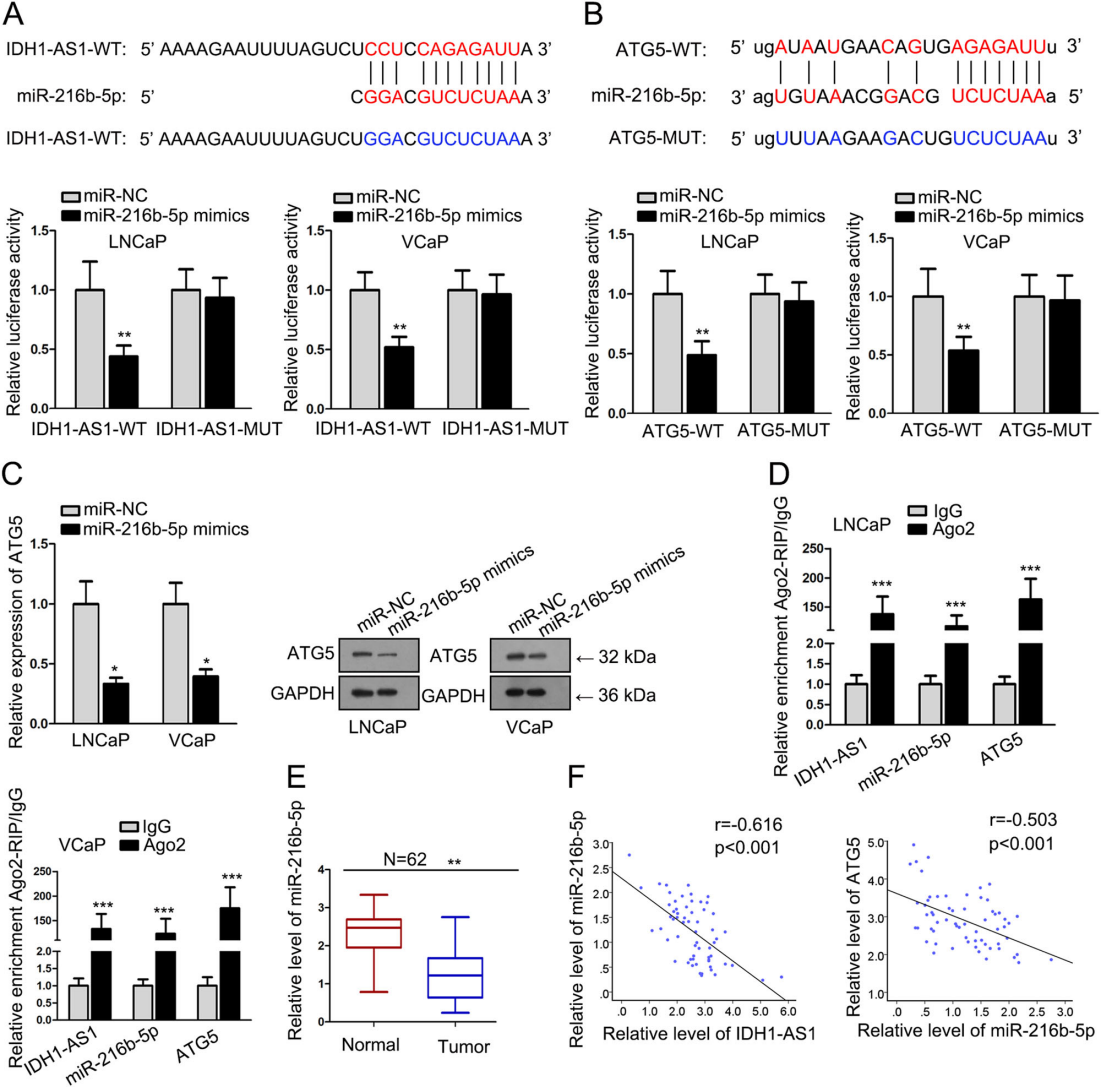
IDH1-AS1 upregulated ATG5 expression by sponging miR-216b-5p. **a**, **b** Wild type or mutant type of IDH1-AS1 or ATG5 3′UTR containing the binding sequences with miR-216b-5p was subjected to luciferase reporter assay. **c** The mRNA and protein level of ATG5 in cells transfected with miR-NC or miR-216b-5p mimics. **d** Ago2-RIP assay was used to prove the enrichment of IDH1-AS1, miR-216b-5p and ATG5 in RISC complex. **e** miR-216b-5p expression was measured in PCa tissues than that in normal controls. **f** Pearson correlation analysis was applied to evaluate expression correlation between miR-216b-5p and IDH1-AS1 or ATG5. *P < 0.05, **P < 0.01, ***P < 0.001

CeRNA regulatory network is an important posttranscriptional regulatory mechanism of lncRNAs. Here, we also explored whether IDH1-AS1 and ATG5 could form a ceRNA pathway. Bioinformatics analysis suggested that miR-216b-5p had complementary base pairing with both IDH1-AS1 and ATG5. To validate their interaction, IDH1-AS1 or ATG5 3′UTR containing the binding sequences with miR-216b-5p was cloned into luciferase reporter vectors. It was uncovered that the luciferase activity of wild type reporters was decreased by miR-216b-5p mimics, whereas no obvious changes in mutant reporters (Fig. 6a, b). Then, we examined the mRNA and protein level of ATG5 in cells transfected with miR-NC or miR-216b-5p mimics. The levels of ATG5 were significantly impaired by the upregulation of miR-216b-5p (Fig. 6c). Ago2-RIP assay was used to prove the enrichment of IDH1-AS1, miR-216b-5p, and ATG5 in RISC complex (Fig. 6d). Afterwards, miR-216b-5p expression was found to be lower in PCa tissues than that in normal controls (Fig. 6e). Pearson correlation analysis revealed that miR-216b-5p had negative expression correlation with IDH1-AS1 and ATG5 (Fig. 6f). All these experimental results indicated that IDH1-AS1 functioned as a ceRNA to regulate ATG5 expression via sponging miR-216b-5p.

### IDH1-AS1 promoted PCa cell growth by regulating ATG5-mediated autophagy

**Fig. 7:**
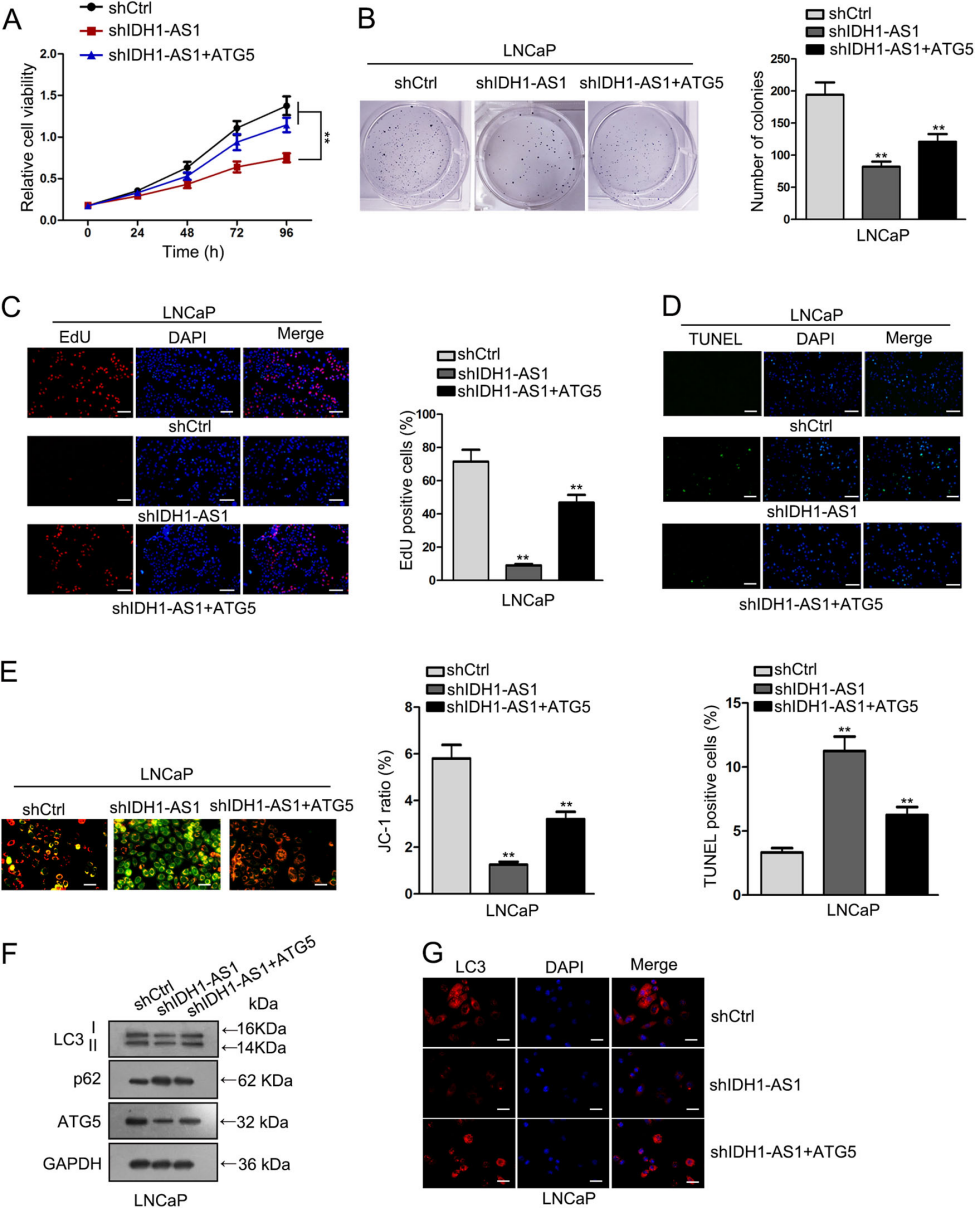
IDH1-AS1 promoted PCa cell growth by regulating ATG5-mediated autophagy. The involvement of ATG5 in IDH1-AS1-mediated cell proliferation was determined by CCK-8 (**a**), colony formation (**b**) and EdU assays (**c**). Apoptosis was measured in indicated PCa cells through JC-1 (**d**) and TUNEL assays (**e**). **f**, **g** Changes in autophagy were observed in indicated PCa cells by western blotting and LC3 immunofluorescence test. **P < 0.01. Scale bars for EdU, TUNEL, JC-1 assays and LC3 immunofluorescence equal 200 μm

**Fig. 8:**
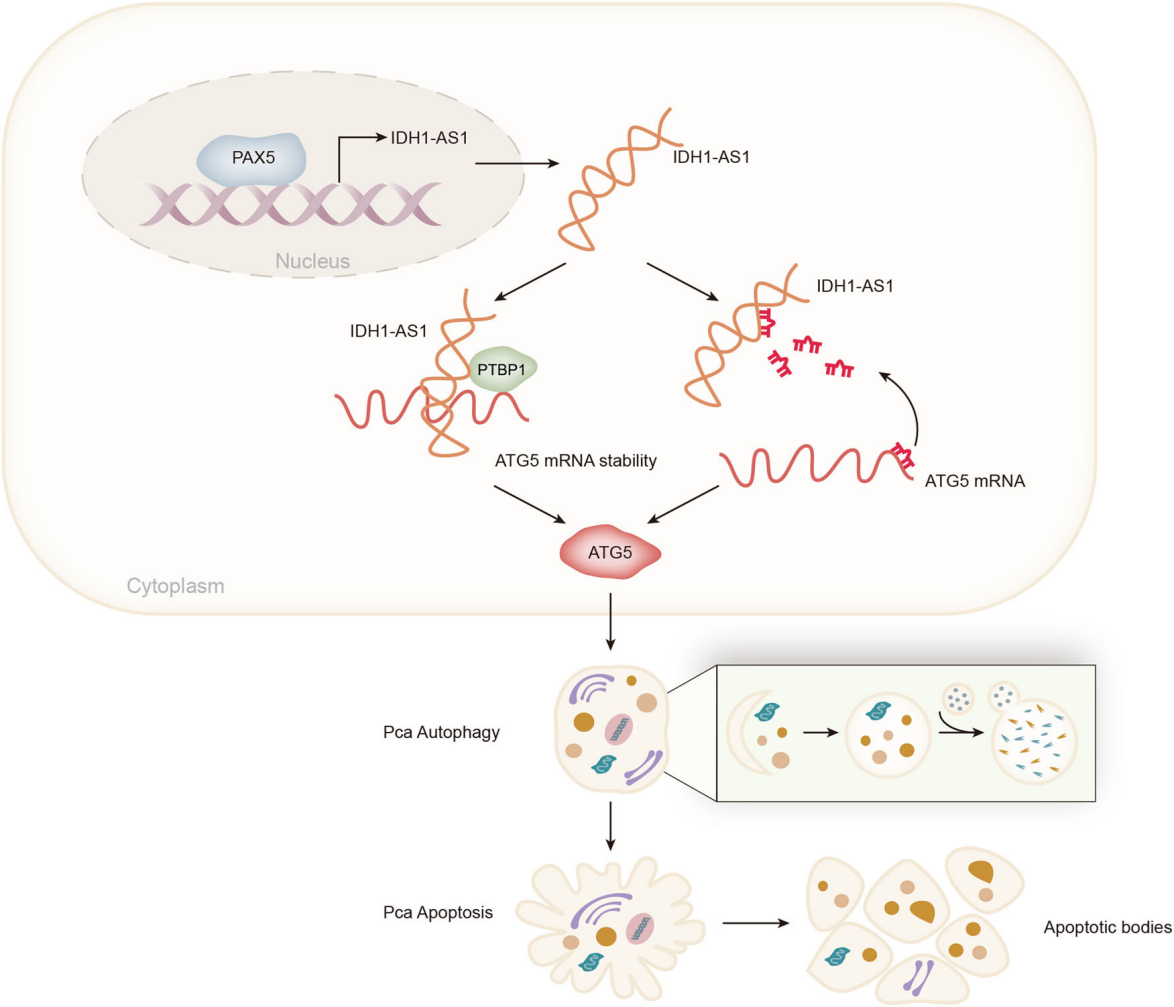
Graphical abstract was plotted to illustrate the molecular mechanism of IDH1-AS1 in PCa tumor growth

To demonstrate IDH1-AS1 promoted PCa cell growth via ATG5, rescue assays were carried out in LNCaP cells. Through cell proliferation assay, we determined that co-transfection with ATG5 expression vector rescued shIDH1-AS1-induced proliferation-inhibition (Fig. 7a–c). Moreover, shIDH1-AS1-induced apoptosis was reduced after overexpression of ATG5 (Fig. 7d, e). Simultaneously, changes in autophagy were observed. It was showed that ATG5 overexpression recovered the autophagy suppressed by IDH1-AS1 knockdown (Fig. 7f, g). Combining with all experimental data above, PAX5-activated IDH1-AS1 acted as an oncogene in PCa tumor growth by promoting ATG5-induced autophagy (Fig. 8).

## Discussion

Recent years, lncRNAs have been elucidated in various reports due to their biological functions^21–23^. In addition, dysregulation of lncRNAs is acknowledged as an important factor for tumorigenesis^24–26^. The key finding of current study is that IDH1-AS1 was upregulated in PCa tissues and cells. In our current study, we investigated whether IDH1-AS1 is a regulator in PCa tumorigenesis. Through loss-of function assays, we determined that IDH1-AS1 had a positive effect on PCa cell proliferation but a negative effect on PCa cell apoptosis. Moreover, animal study was carried out and proved that IDH1-AS1 was a facilitator for PCa tumor growth. Therefore, oncogenic property of IDH1-AS1 in PCa tumorigenesis was identified in this study.

Transcription activation is a reason for the upregulation of lncRNAs in tumorigenesis^11–13^. In this study, we analyzed the upstream molecular mechanism of IDH1-AS1. Through mechanism investigation, PAX5 was verified to be a transcription activator for IDH1-AS1 and positively regulated IDH1-AS1 expression in PCa. Downstream molecular mechanism of IDH1-AS1 in PCa was further investigated. Through microarray analysis and GO analysis, we uncovered that IDH1-AS1 potentially regulated autophagy in PCa cells via modulating ATG5 expression. According to the experimental results, we determined that IDH1-AS1 promoted autophagy in PCa cells by upregulating ATG5.

Based on all findings above, we continued to explore whether IDH1-AS1 exerts functions by regulating ATG5 expression. Luciferase activity of ATG5 promoter was not affected by IDH1-AS1 and the cytoplasmic localization of IDH1-AS1 prompted us to investigate posttranscriptional regulation of IDH1-AS1 on ATG5. There are two regulatory patterns of lncRNAs at post-transcriptional level. For example, lncRNAs can regulate mRNA stability by interacting with RBPs^17–20^; lncRNAs positively regulate mRNAs by sequestering miRNAs^14–16^. At first, pull-down silver staining followed by mass-spectrometry analysis revealed that there were some RBPs that could interact with IDH1-AS1. Mechanism investigation showing the interactions between IDH1-AS1 and PTBP3 as well as between PTBP3 and ATG5. Furthermore, we found that IDH1-AS1 enhanced the stability of ATG5 mRNA by cooperating with PTBP3. Subsequently, we investigated whether IDH1-AS1 acts as a ceRNA to regulate ATG5 in PCa cells. Similarly, mechanism study demonstrated that IDH1-AS1 acted as a ceRNA to upregulate ATG5 expression by sequestering miR-216b-5p. At length, we analyzed the involvement of ATG5 in IDH1-AS1-mediated cell growth and autophagy by rescue assays. It was uncovered that cell growth and autophagy that were suppressed by IDH1-AS1 knockdown were recovered by the overexpression of ATG5. Taken together, we confirmed that IDH1-AS1 acted as a growth facilitator in PCa cells via regulating ATG5-mediated autophagy.

In conclusion, this study reported a novel molecular pathway in PCa tumorigenesis. Upregulation of IDH1-AS1 was induced by PAX5 transcription activator. Moreover, upregulation of IDH1-AS1 facilitated PCa tumor growth by posttranscriptionally regulating ATG5 expression. All findings in this study may contribute to unveil molecular mechanism associated with tumorigenesis of PCa, thus providing new potential diagnostic or therapeutic biomarkers.

## Supplementary information

Figure S1

Supplementary figure legends
