# Supplementary figures and images for: PAX5-induced upregulation of IDH1-AS1 promotes tumor growth in prostate cancer by regulating ATG5-mediated autophagy
Source: Cell Death Dis. 2019 Sep 30;10(10):734. doi: 10.1038/s41419-019-1932-3 (PMC6769014; doi:10.1038/s41419-019-1932-3)

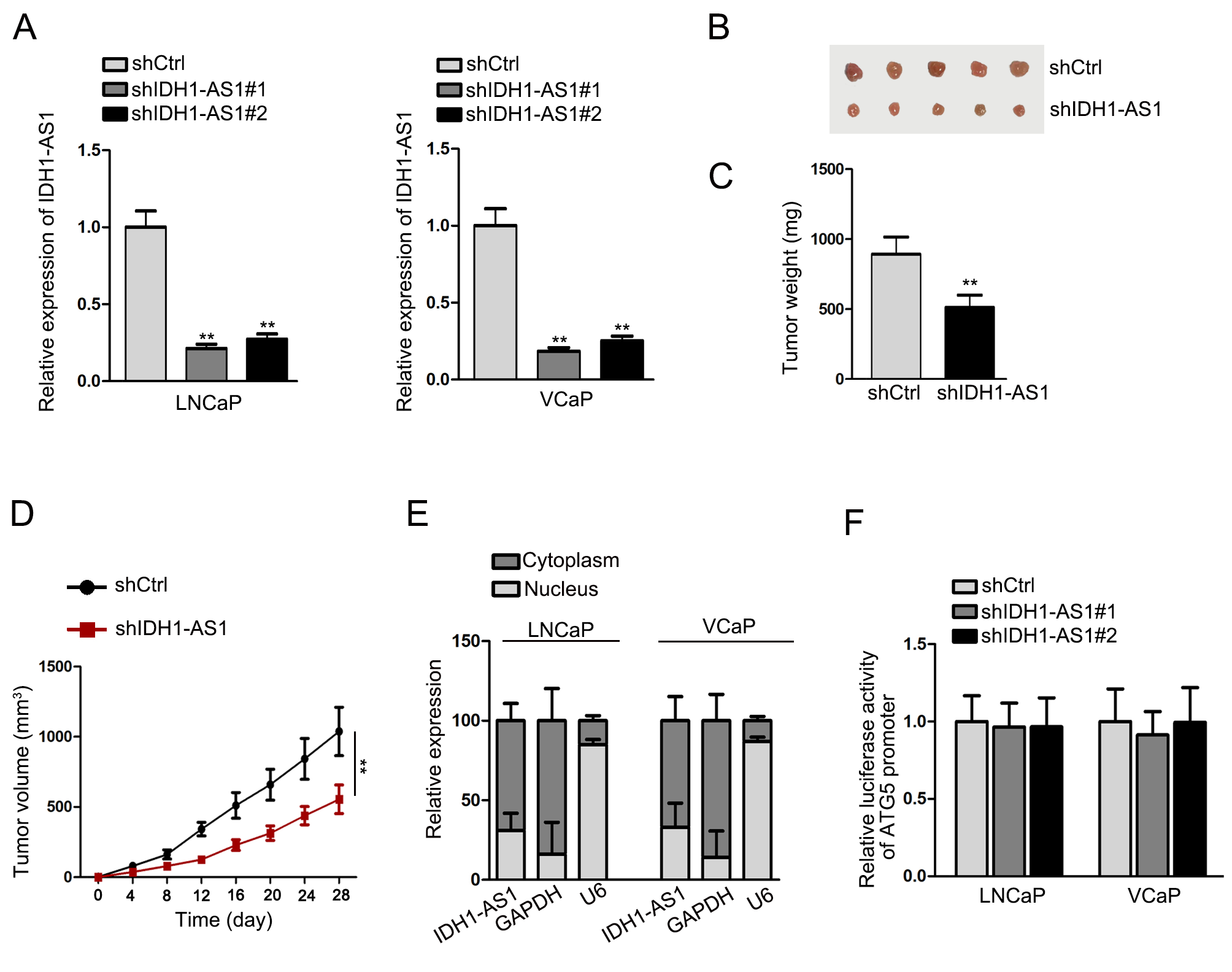

Supplement: Supplementary file 1 — Figure S1 [file 41419_2019_1932_MOESM1_ESM.tif]
