# Supplementary material for: PAX5-induced upregulation of IDH1-AS1 promotes tumor growth in prostate cancer by regulating ATG5-mediated autophagy
Source: Cell Death Dis. 2019 Sep 30;10(10):734. doi: 10.1038/s41419-019-1932-3 (PMC6769014; doi:10.1038/s41419-019-1932-3)
Supplement: Supplementary file 2 — Supplementary figure legends [file 41419_2019_1932_MOESM2_ESM.docx]

**Supplementary Figure 1.** (A) Silencing of IDH1-AS1 by shRNAs was determined by qRT-PCR analysis. (B) After 28 days, tumors were removed from the body of nude mice treated with transfected cells were listed. (C-D) Tendencies in tumor volume and weight were measured. (E) Cellular localization of IDH1-AS1 was assessed with subcellular fractionation assay. (F) pGL3 luciferase reporter assay was conducted to detect the effect of IDH1-AS1 on the luciferase activity of ATG5 promoter. ^**^P < 0.01.
